# Long-Term Net Harvesting Is Associated with Elevated Soil Carbon Pool Management Index in Chinese Hickory Plantations

**DOI:** 10.3390/plants15172589

**Published:** 2026-08-25

**Authors:** Jiale Zhou, Yinglei Huang, Manting Yang, Wei Dai, Jin Jin, Weijun Fu

**Affiliations:** 1 National Key Laboratory for Development and Utilization of Forest Food Resources, Zhejiang A&F University, Hangzhou 311300, China; jlzhou@stu.zafu.edu.cn (J.Z.); ymt0011934@yeah.net (M.Y.); jinjindh1995@163.com (J.J.); 2 College of Environment and Resources (College of Carbon Neutrality), Zhejiang A&F University, Hangzhou 311300, China; 3 Administration of Zhejiang Qingliangfeng National Nature Reserve, Hangzhou 311300, China; gaodm_1@163.com; 4 Department of Biosystems Engineering and Soil Science, University of Tennessee, Knoxville, TN 37996, USA; daiweiaq180@gmail.com

**Keywords:** soil labile organic fraction, enzyme activity, soil microbial community, understory vegetation, carbon cycling

## Abstract

As the unique edible nut species in China, Chinese hickory (*Carya cathayensis* Sarg.) is vulnerable to anthropogenic disturbances that reduce vegetation residue inputs and soil organic matter accumulation. To investigate the effects of net-harvesting-associated understory management on the soil carbon pool management index (CPMI), soil samples were collected from *C. cathayensis* plantations subjected to net harvesting for 2, 3, 6, and 7 years, with traditional beating as the control. Long-term net harvesting significantly increased particulate and dissolved organic carbon, microbial biomass carbon, and labile organic carbon (*p* < 0.05). It also altered soil enzyme activities, microbial community composition, and microbial co-occurrence network complexity. Soil physical and chemical properties and carbon pools were key drivers of enzyme activities and microbial complexity. Partial least squares path modeling (PLS-PM) revealed that understory vegetation positively influenced the CPMI (total effect = 0.57), while soil biological processes also contributed to CPMI variation. Overall, net harvesting reduced anthropogenic disturbance, enhanced litter-derived carbon inputs, and promoted soil biological functions, thereby improving the CPMI. These findings provide insights into sustainable soil management and carbon sequestration in *C. cathayensis* plantations.

## 1. Introduction

Climate change driven by increasing atmospheric CO_2_ concentrations poses a major global challenge [1,2]. Soil organic carbon (SOC), representing the organic carbon stored in soils, is a key component of terrestrial carbon pools and plays an essential role in regulating soil fertility and ecosystem carbon cycling. However, carbon dynamics in managed plantations may differ from those in natural forests because of differences in species composition and human disturbance. Based on turnover rates, the SOC pool can be divided into passive and active carbon pools [3]. The passive carbon pool, affected through physical occlusion and biochemical recalcitrance, shows slow turnover rates and limited sensitivity to short-term soil carbon dynamics [4,5]. In contrast, the active carbon pool, referred to as the labile carbon pool, primarily comprises dissolved organic carbon (DOC), microbial biomass carbon (MBC), particulate organic carbon (POC), and permanganate oxidizable carbon (POXC) [6]. The active carbon pool can facilitate soil nutrient cycling due to its rapid oxidation, mineralization, and decomposition rate [7]. The carbon pool management index (CPMI), calculated based on SOC and POXC, serves as a sensitive indicator of SOC turnover dynamics [8,9]. It is also an effective parameter for soil quality evaluation that reflects quantitative changes in labile organic matter. Consequently, the CPMI offers a comprehensive assessment of soil quality degradation or restoration resulting from land-use changes or anthropogenic management practices [10].

SOC sequestration is influenced by multiple factors, including regional climate, soil properties, root biomass, and anthropogenic management practices (e.g., fertilization, straw return, and conservation tillage) [11,12,13,14]. At the same time, studies have demonstrated that increased SOC content could enhance the availability of soil nutrients, water-holding capacity, and porosity [15,16]. Furthermore, there are strong correlations between SOC and the composition and diversity of soil microbial communities [17]. Higher SOC usually promotes soil buffering capacity and microbial diversity to improve soil health and agricultural productivity [18]. Elucidating the driving mechanisms of different influencing factors on SOC dynamics is fundamental to understanding soil–atmosphere carbon cycling, and it is crucial for accurately predicting the contribution of SOC dynamics to terrestrial carbon sequestration. Therefore, identifying appropriate management practices is of paramount importance for enhancing SOC sequestration and mitigating greenhouse gas emissions.

As important managed tree-based production systems in China, economic forests provide essential food and economic products while reducing pressure on natural forests through a substitution effect. By supplying commercially valuable products such as nuts and woody oils, these plantations can partially replace resources obtained from natural forests and contribute to regional ecological sustainability [19]. However, the absence of well-established management protocols and integrated policy support leads to the neglect of ecological sustainability in some Chinese economic forests. However, the absence of well-established management protocols and integrated policy support has resulted in insufficient consideration of ecological sustainability in some Chinese economic forests [20]. For example, intensive monoculture management and frequent soil disturbances in economic plantations can exacerbate ecological vulnerabilities, resulting in declining soil fertility, degraded soil structure, and reduced stand productivity [21]. Furthermore, soil structure degradation caused by water erosion and frequent anthropogenic activities has resulted in deteriorated soil physical properties and significantly diminished water retention capacity [22]. Therefore, there is an urgent need to develop and widely implement ecological management practices that can effectively improve both the environmental quality and productivity of economic forests.

Chinese hickory (*Carya cathayensis* Sarg.), a perennial deciduous tree species of Juglandaceae and Carya, is an endemic premium nut and woody oil crop in China [23]. Traditional management practice such as herbicides application was used to remove understory vegetation. Moreover, direct beating with bamboo poles was used to collect Chinese hickory nuts. These practices have led to the total removal of shrubs and herbaceous plants and an expanded area of bare soil, conditions that hinder soil and water conservation [24]. This traditional practice involves repeated striking of branches, bark damage, leaf loss, and soil compaction due to human trampling. These practices may increase soil disturbance and reduce understory vegetation coverage, potentially affecting soil conservation functions and soil quality in hickory plantations. To mitigate soil degradation, it is necessary to introduce net harvesting technology to collect naturally mature nuts [25]. This technique is associated with reduced herbicide dependence and greater persistence of understory vegetation, which may contribute to improved soil ecological functions. It simultaneously minimizes mechanical damage to fruits and reduces reliance on herbicides, thereby conserving understory vegetation and boosting biodiversity. However, the specific path of how net harvesting-associated management regimes are related to soil carbon pool dynamics by changing understory vegetation remains unclear. Therefore, this study can supplement the gaps in this type of correlation. We hypothesized that prolonged net harvesting-associated management would be associated with a higher carbon pool management index (CPMI) and shifts in microbial community characteristics through enhanced understory vegetation recovery and reduced soil disturbance. Our study comparatively analyzed *C. cathayensis* stands under varying net harvesting durations against traditional pole-harvesting controls. To establish the theoretical framework for carbon pool regulation in economic forests, we investigated how understory vegetation dynamics, soil labile organic carbon fractions, enzyme activities, and microbial network complexity responded to net harvesting, as well as how these factors interacted to affect the CPMI.

## 2. Results

### 2.1. Effects of Net-Harvesting Duration on Aboveground Vegetation

Significant differences in vegetation biomass (VB), vegetation total carbon (VC), and vegetation total carbon-to-total nitrogen ratio (VC/VN) were observed among the five net harvesting-associated management (CK, Y2, Y3, Y6, and Y7, representing 0, 2, 3, 6, and 7 years of net harvesting, respectively) in the *C. cathayensis* plantations (*p* < 0.05) (Table 1). In contrast, vegetation total nitrogen (VN) showed no significant variation across all treatments. The results demonstrated that prolonged net-harvesting management significantly increased VB (*p* < 0.05). Compared with CK, VB increased by 364.52% and 292.88% under Y6 and Y7 treatments, respectively, and Y3 showed significantly higher VC than CK (*p* < 0.05). Notably, Y3 exhibited the maximum VC/VN ratio, which was significantly greater than CK (*p* < 0.05).

**Figure 1:**
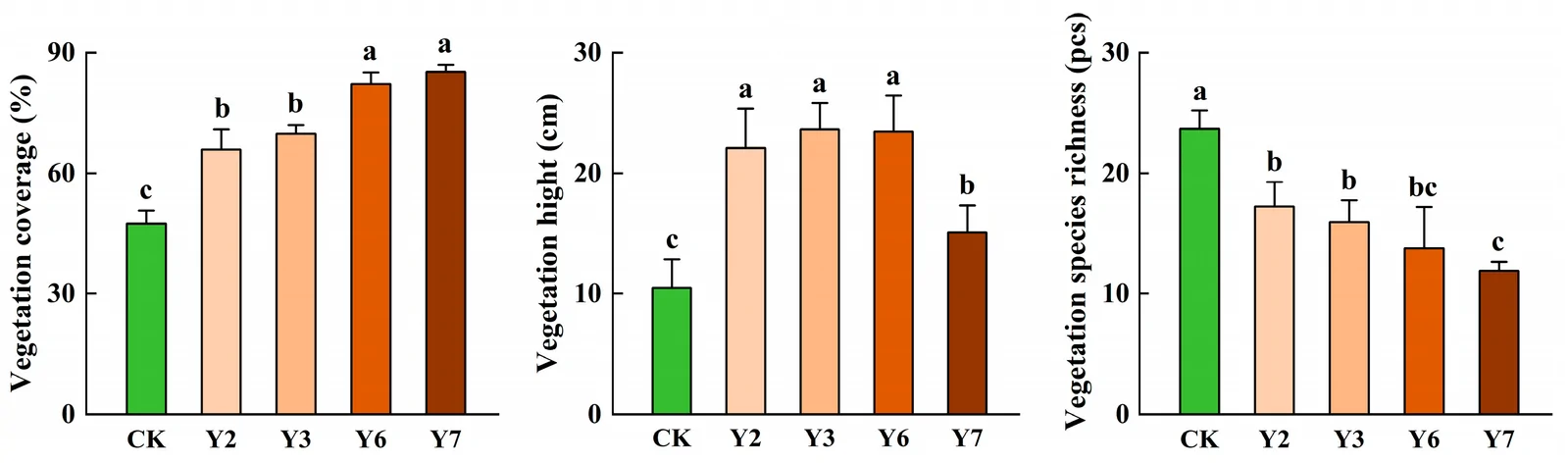
Vegetation characteristics under different treatments in *C. cathayensis* plantations. Different letters denote significant differences (*p* < 0.05).

Vegetation coverage, height, and species richness displayed significant variations under different net-harvesting regimes (*p* < 0.05, Figure 1). Vegetation species richness was calculated based on the plot survey data using the richness index, the Shannon–Wiener index, and the Simpson index according to Equations (1)–(3). The extended net-harvesting duration substantially enhanced vegetation coverage (*p* < 0.05), exceeding 80% in Y6, while it was lower than 50% in CK. Compared with CK, vegetation height increased by 110.91% in Y2, but significantly decreased in Y7 (*p* < 0.05). Conversely, species richness showed a considerable declining trend with increasing net-harvesting duration (*p* < 0.05). Net harvesting altered understory vegetation composition, with Y6 showing greater community complexity compared with CK (Table S1). The relative abundance of the primary dominant species initially decreased, then increased with extended net harvesting. Furthermore, the understory vegetation diversity indices showed differences among net-harvesting durations, with the Shannon–Wiener and Simpson indices varying among treatments (Table S2).

### 2.2. Effects of Net-Harvesting Duration on Soil Quality

Net-harvesting duration significantly altered soil physicochemical properties in *C. cathayensis* plantations (*p* < 0.05; Table S3), with treatment differences presented in Table 2. Soil pH was significantly affected by net-harvesting duration and season (*p* < 0.01; Table S3). In summer, bulk density (BD) was significantly higher in CK and Y2 than in the other treatments (*p* < 0.05). Compared with winter, BD showed higher values in summer, particularly in CK and Y2. In terms of soil nutrients, the available nitrogen (AN) content increased significantly with net-harvesting duration in summer (*p* < 0.05), with the peak value detected under the Y6 treatment. Conversely, the AN content was significantly higher in CK and Y3 (*p* < 0.05) in winter. In summer, the contents of available phosphorus (AP) and available potassium (AK) were the highest in Y7. The AP content decreased significantly with net-harvesting duration in winter, while the AK content showed an opposite trend (*p* < 0.05). Thus, net harvesting generally enhanced soil nutrient availability, with higher summer values than winter.

Net harvesting significantly affected SOC, TN, and the C/N ratio (*p* < 0.05; Table 3 and Table S3). Specifically, SOC was significantly affected by treatment, whereas TN showed significant seasonal effects. The C/N ratio was significantly affected by both treatment and season (Table S3). The SOC content increased significantly with net-harvesting duration (*p* < 0.05), with the highest values observed under the Y6 treatment during both summer and winter. In summer, the TN content was significantly higher in Y6 and Y7 (*p* < 0.05). All C/N ratios remained below 25 but showed an increasing trend with net-harvesting duration (*p* < 0.05).

### 2.3. Changes in Soil Carbon Pool Under Different Net-Harvesting Durations

**Figure 2:**
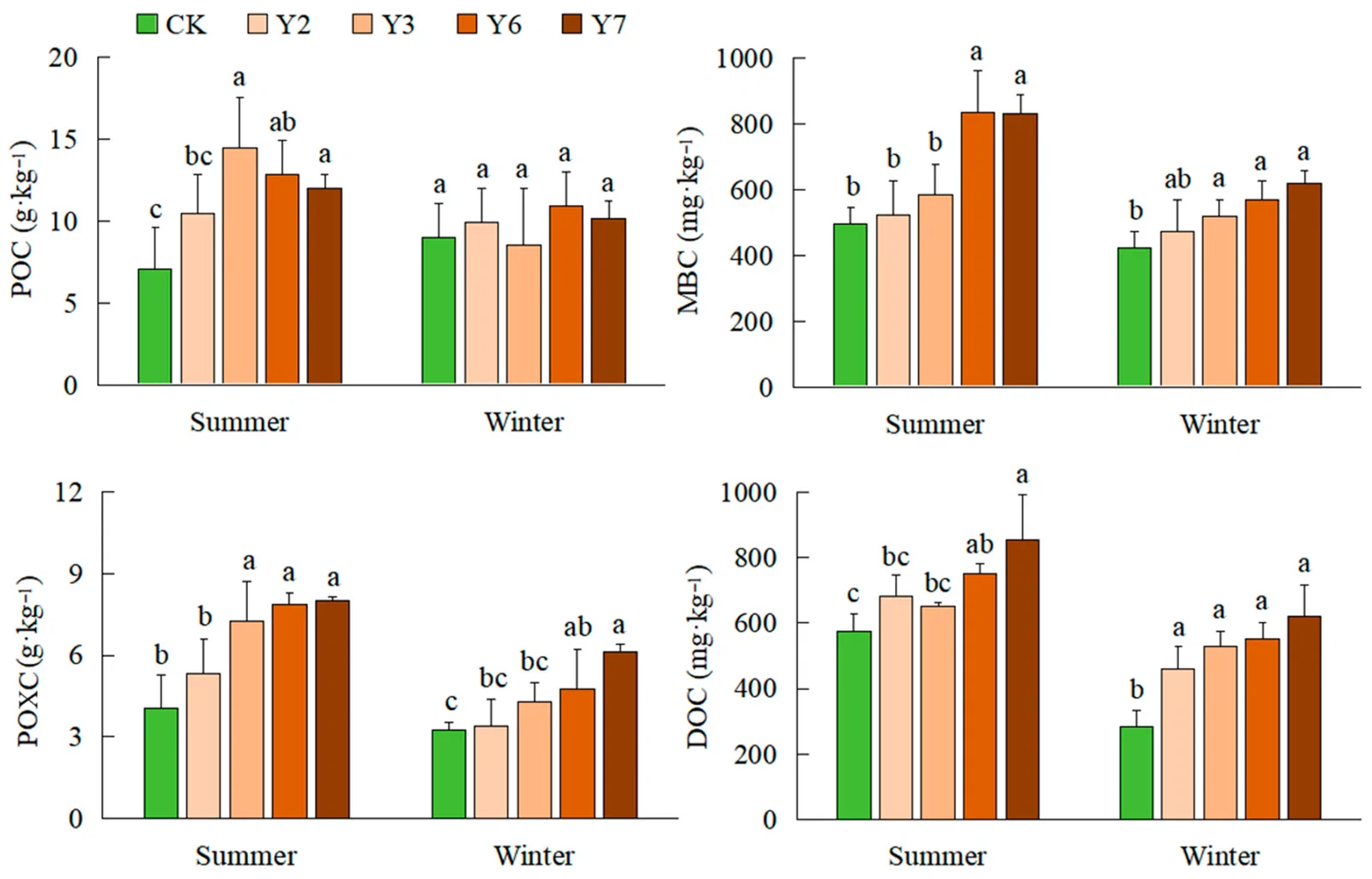
Contents of soil labile organic carbon fractions under different treatments. Different letters denote significant differences (*p* < 0.05).

Labile organic carbon fractions were generally higher in summer than in winter and differed significantly between traditional pole-harvesting and net-harvesting treatments (*p* < 0.05; Figure 2). Specifically, DOC, MBC, and POXC showed highly significant treatment and seasonal effects (Table S3, *p* < 0.001). In summer, the POC and MBC contents also showed significant differences among treatments (*p* < 0.05). Specifically, the POC content initially increased and then decreased with increasing net-harvesting duration, reaching the highest value in Y3. In contrast, the MBC content was significantly higher in Y6 and Y7 than at other durations (*p* < 0.05). Compared with CK, net harvesting obviously enhanced the POXC and DOC contents (*p* < 0.05). Specifically, the DOC content in Y7 increased by 40.62% in summer (*p* < 0.05). Linear regression analysis revealed that POC was substantially positively correlated with SOC (*p* < 0.05; Figure S1), while POXC, MBC, and DOC exhibited highly significant positive correlations with SOC (*p* < 0.001).

During summer, the carbon pool index (CPI) increased significantly by 28.00% to 167.00% with prolonged net-harvesting duration (*p* < 0.05; Table 4). In contrast, L decreased significantly in Y6 and Y7 compared with CK (*p* < 0.05; Table 4). In winter, LI showed a lower value in Y2 compared with other treatments, while exhibiting the highest value in Y7. Overall, the carbon pool management index (CPMI) exhibited significant variations among the net-harvesting treatments (Table S3), with peak values observed in the Y7 treatment for both summer and winter seasons (*p* < 0.05; Table 4).

### 2.4. Soil Enzyme Activities Under Different Net-Harvesting Durations

**Figure 3:**
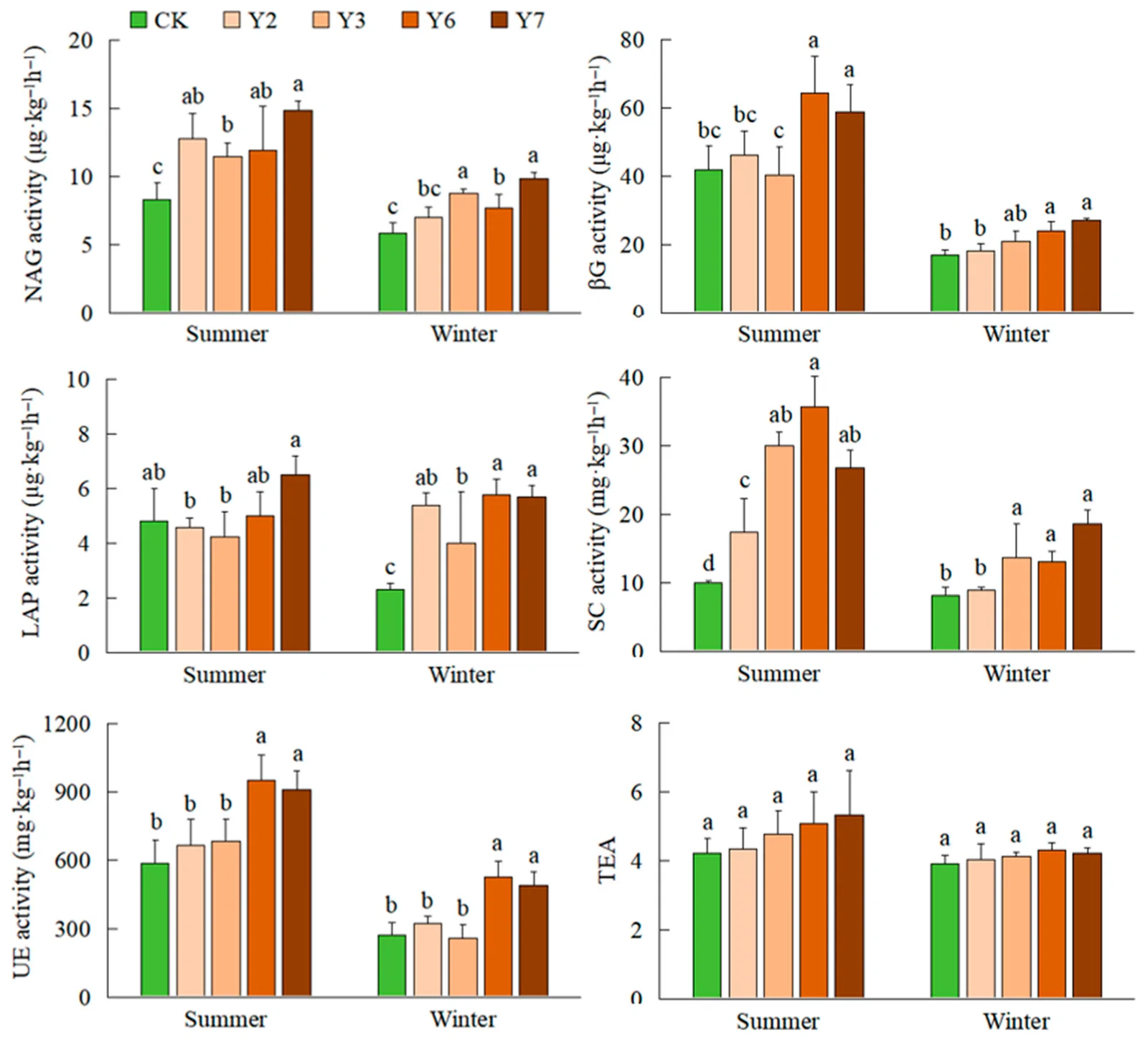
Soil enzyme activity under different treatments. Different letters denote significant differences (*p* < 0.05). TEA: total enzyme activity, which was integrated by summing the normalized ratio ($x_{i}/\bar{x}$) of each enzyme to unify different measurement units of the five soil enzymes (see Formula (9) for calculation details).

Soil enzyme activities were significantly influenced by net harvesting (*p* < 0.05; Figure 3), with generally higher activities observed in summer than in winter. Compared with CK, net harvesting significantly increased the β-1,4-N-acetylglucosaminidase (NAG) activity, reaching its peak at Y7 (*p* < 0.05). During summer, the β-glucosidase (βG) activity was significantly elevated in Y6 and Y7 relative to the other durations (*p* < 0.05). In winter, the βG activity showed a progressive increase with increasing net-harvesting duration (*p* < 0.05). The leucine aminopeptidase (LAP) activity initially decreased and then increased with duration in summer. In winter, it was consistently higher in the net-harvesting treatment than in CK (*p* < 0.05). The sucrase (SC) activity demonstrated obvious seasonal variations: in summer, it increased progressively with net-harvesting duration, peaking in Y6 (*p* < 0.05), whereas in winter, its activity in Y7 surpassed that in the other treatments (*p* < 0.05). The urease (UE) activity was maximized in Y6 compared with the other treatments (*p* < 0.05).

### 2.5. Characteristics of Soil Microbial Community Structure Under Different Netting Durations

#### 2.5.1. Changes in Soil Bacterial Communities Under Different Netting Harvest Years

After quality filtering, denoising, paired-end merging, and chimera removal using the DADA2 pipeline, the number of non-chimeric sequences retained for downstream analysis ranged from 70,024 to 91,057 reads per sample. The observed ASV numbers ranged from 1891 to 2630 among samples. Rarefaction curves approached asymptotic plateaus with increasing sequencing depth, indicating that the sequencing depth was sufficient to capture the majority of microbial diversity and was comparable among samples (Figure S2).

**Figure 4:**
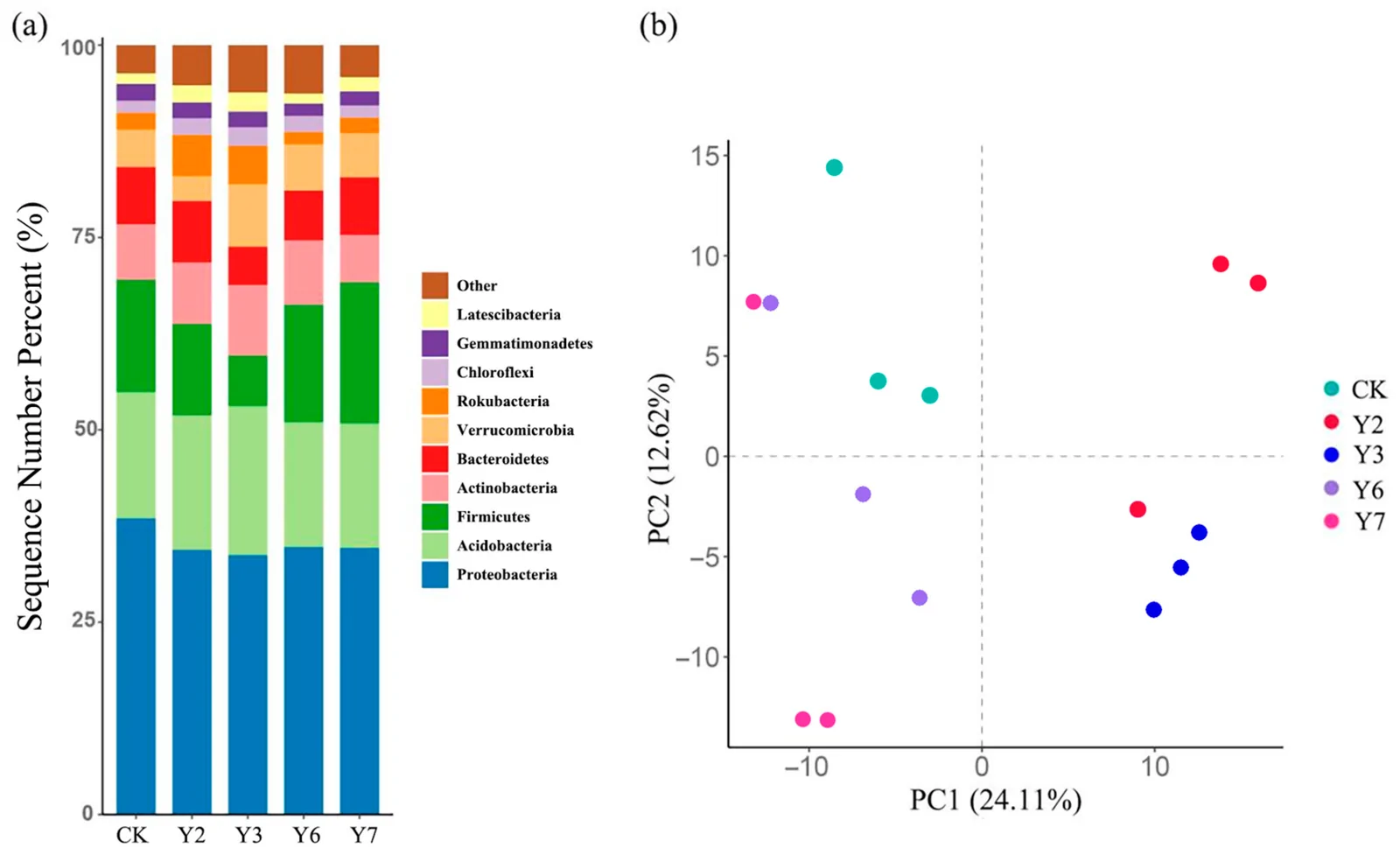
Soil bacterial phylum composition (**a**) and PCA analysis (**b**) under different treatments.

Analysis of soil bacterial composition in *C. cathayensis* plantations under different netting durations (Figure 4) revealed that the top 10 most abundant phyla in surface soils were dominated by *Proteobacteria* (33.73–38.50%), *Acidobacteria* (16.16–19.36%), and *Firmicutes* (6.59–18.37%) at the phylum level. Compared with CK, net harvesting increased the *Proteobacteria* abundance by 0.68–4.77%. CK showed the highest *Acidobacteria* abundance (19.36%), which decreased with increasing netting duration (Figure 4a). In contrast, the *Firmicutes* abundance increased with netting duration, peaking at 18.37% in Y7 (Figure 4a). Principal component analysis (PCA) based on phylum-level relative abundance data showed variation patterns in bacterial community composition among treatments, with PC1 and PC2 explaining 24.11% and 12.62% of the total variation, respectively (Figure 4b).

**Figure 5:**
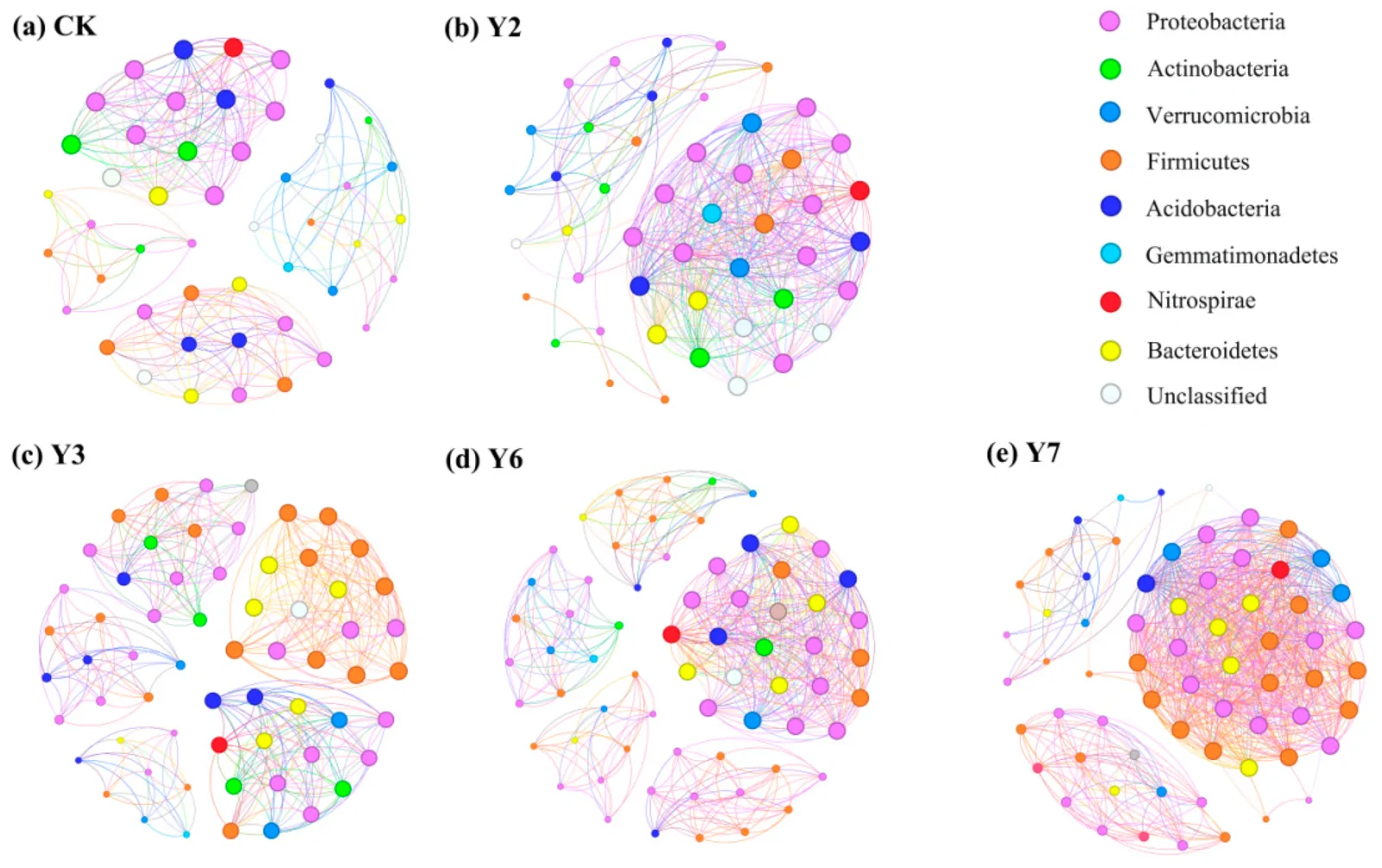
Network visualization of soil bacterial communities under different treatments. (**a**) CK treatment; (**b**) Y2 treatment; (**c**) Y3 treatment; (**d**) Y6 treatment; (**e**) Y7 treatment. Each node represents a species or taxon, with edges indicating significant correlations between them (*p* < 0.05). The size of each node is proportional to the relative abundance of the taxon, while the width of each connection corresponds to the Spearman correlation coefficient. Red lines denote positive correlations, whereas green lines represent negative correlations. Other edge colors are only for visual differentiation. The same applies below.

Ecological co-occurrence networks were constructed based on soil bacterial sequencing data (Figure 5, Table 5). The bacterial networks exhibited increased complexity with prolonged net-harvesting duration, characterized by greater numbers of nodes and edges, particularly under the Y6 and Y7 treatments (Table 5). These results indicated that long-term net harvesting may promote more complex bacterial co-occurrence patterns. Compared with CK, the net-harvesting treatments exhibited higher numbers of nodes and connections, accompanied by increases in average degree and graph density, suggesting more complex co-occurrence patterns among bacterial taxa under these management regimes. Y3 showed a relatively higher modularity value than the other treatments (Table 5), indicating a more distinct modular structure within the bacterial network. Positive correlations accounted for the majority of observed associations across all treatments, suggesting prevalent positive co-occurrence patterns among bacterial taxa. Module hub analysis identified *Proteobacteria*, *Acidobacteria*, and *Firmicutes* as important taxa contributing to bacterial network structures (Figure 5).

#### 2.5.2. Changes in Soil Fungal Communities Under Different Net-Harvesting Durations

After quality filtering, denoising, paired-end merging, and chimera removal using the DADA2 pipeline, the number of non-chimeric sequences retained for downstream analysis ranged from 62,401 to 85,491 reads per sample. The observed ASV numbers ranged from 1660 to 2814 among samples. Rarefaction curves approached asymptotic plateaus with increasing sequencing depth, indicating that the sequencing depth was sufficient to capture the majority of microbial diversity and was comparable among samples (Figure S2).

**Figure 6:**
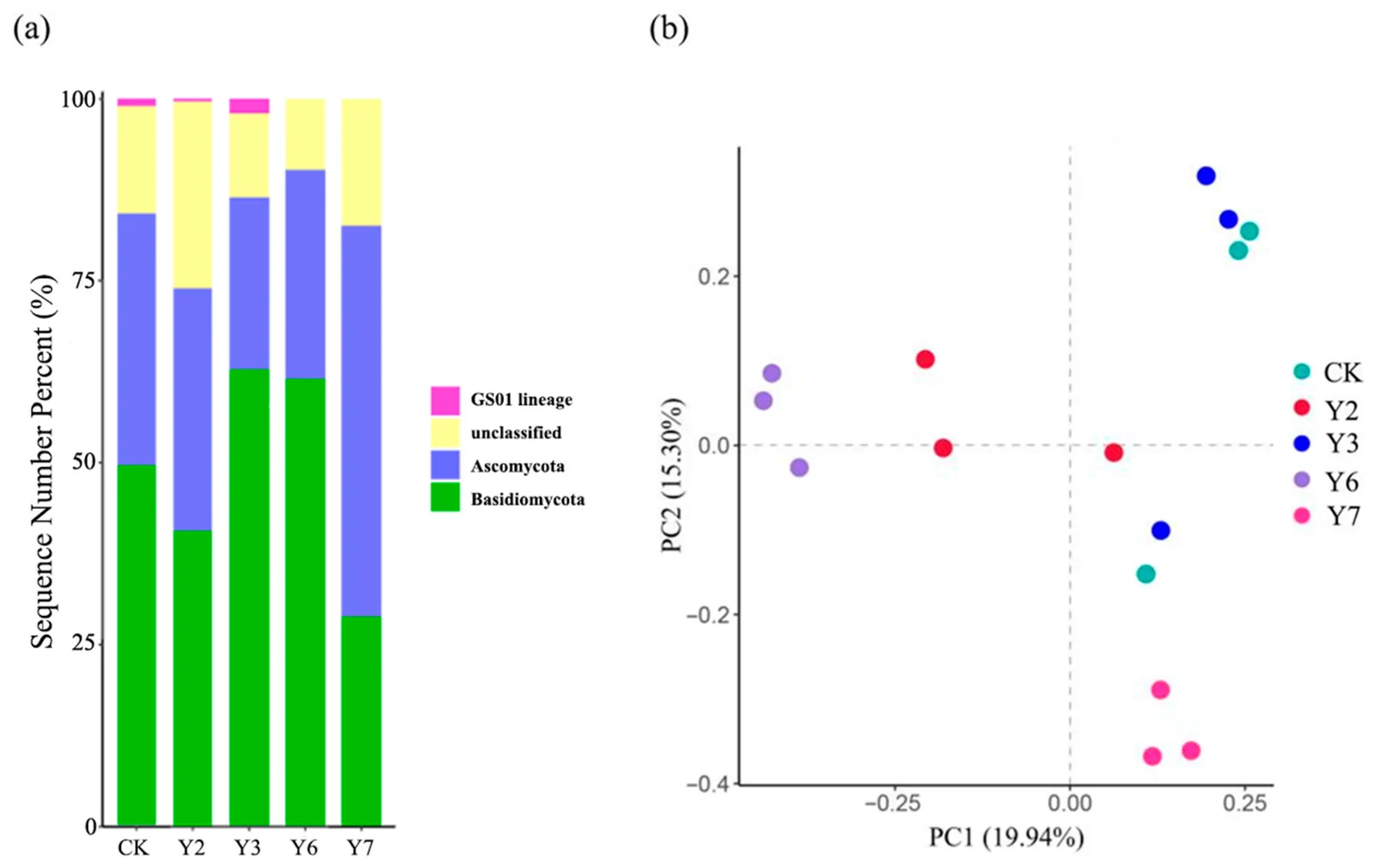
Relative abundance of dominant fungal taxa (**a**) and PCA analysis (**b**) under different treatments.

An analysis of soil fungal composition under different netting durations revealed that the surface soil fungal communities were primarily dominated by *Ascomycota* and *Basidiomycota*, while GS01 represented an unresolved fungal lineage detected across treatments (Figure 6a). Compared with CK, the abundance of *Ascomycota* increased by 19.15% in Y7. The abundance of *Basidiomycota* initially increased and then decreased following netting, reaching a peak (62.81%) in Y3. The highest abundance of GS01 (0.65%) was also observed in Y3. PCA revealed variation patterns in fungal community composition among treatments, with PC1 and PC2 explaining 19.94% and 15.30% of the total variation, respectively (Figure 6b).

**Figure 7:**
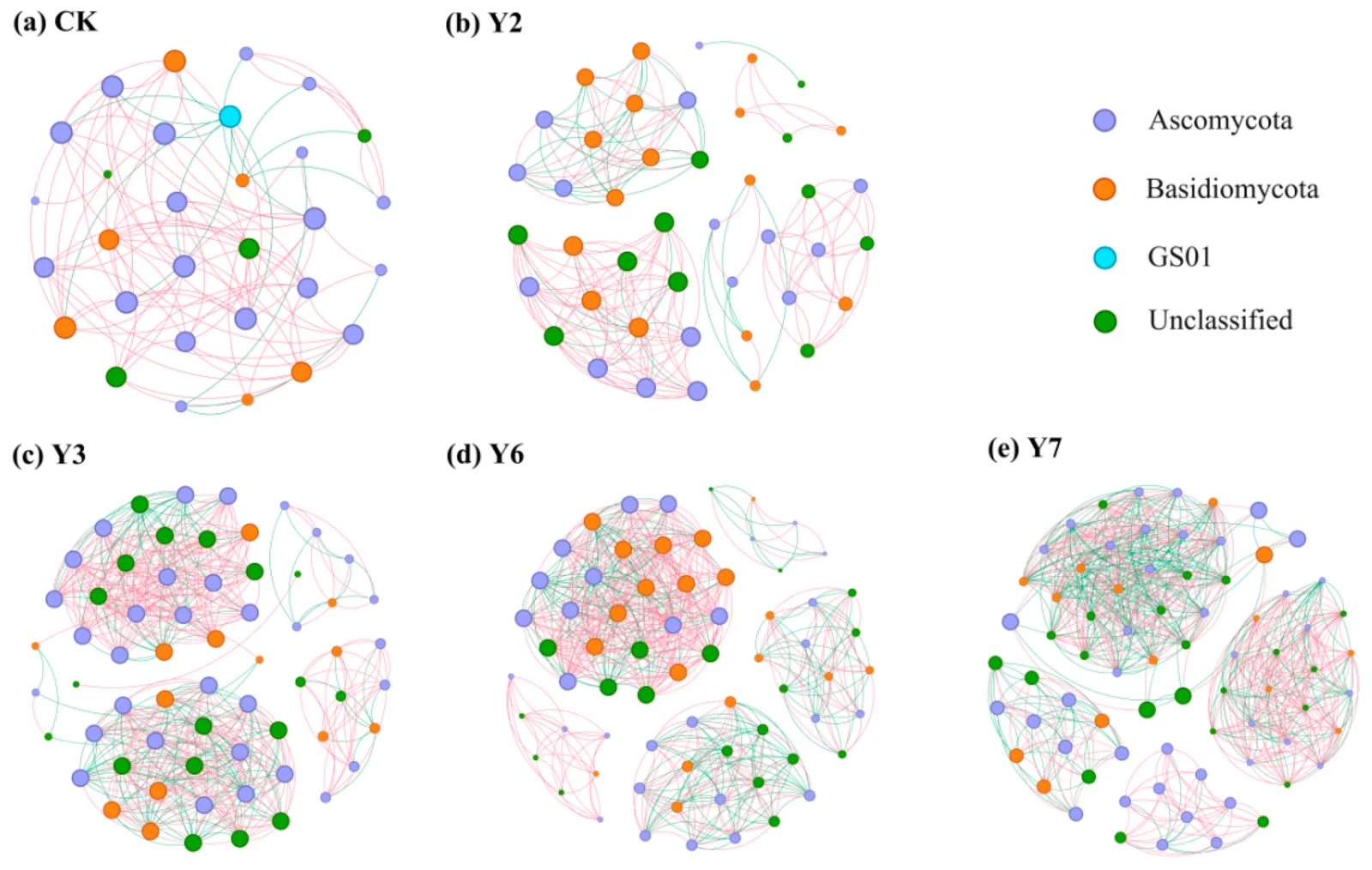
Network visualization of soil fungal communities under different treatments. (**a**) CK treatment; (**b**) Y2 treatment; (**c**) Y3 treatment; (**d**) Y6 treatment; (**e**) Y7 treatment.

Ecological networks were constructed based on fungal sequencing data to explore potential co-occurrence patterns among fungal taxa (Table 6, Figure 7). Compared with CK, net-harvesting treatments increased network size and complexity, as indicated by the higher numbers of nodes, connections, average degree, and graph density (Table 6). These changes suggested that net harvesting may promote more complex fungal association patterns. However, the proportion of positive correlations decreased with increasing net-harvesting duration, indicating shifts in potential fungal interactions among treatments. Module hub analysis identified *Ascomycota*, *Basidiomycota*, and GS01 as keystone taxa governing fungal network topology (Figure 7).

### 2.6. Factors Influencing Soil Carbon Pool Management Index (CPMI)

**Figure 8:**
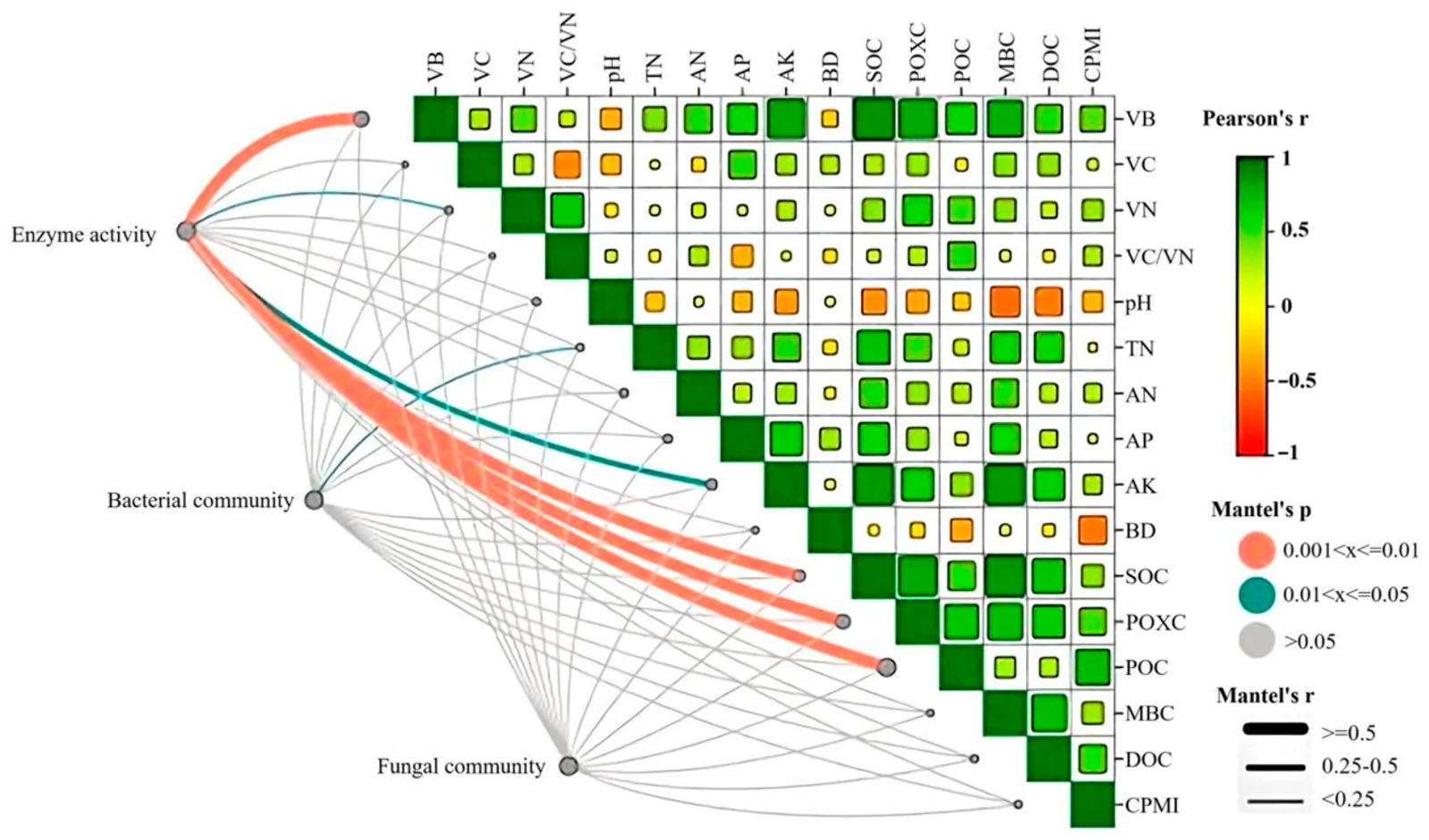
Interactive mantel test correlation heatmap. The edge width represents the Pearson correlation coefficient, while the edge color indicates statistical significance. The pairwise correlations of these variables are displayed using colors.

**Figure 9:**
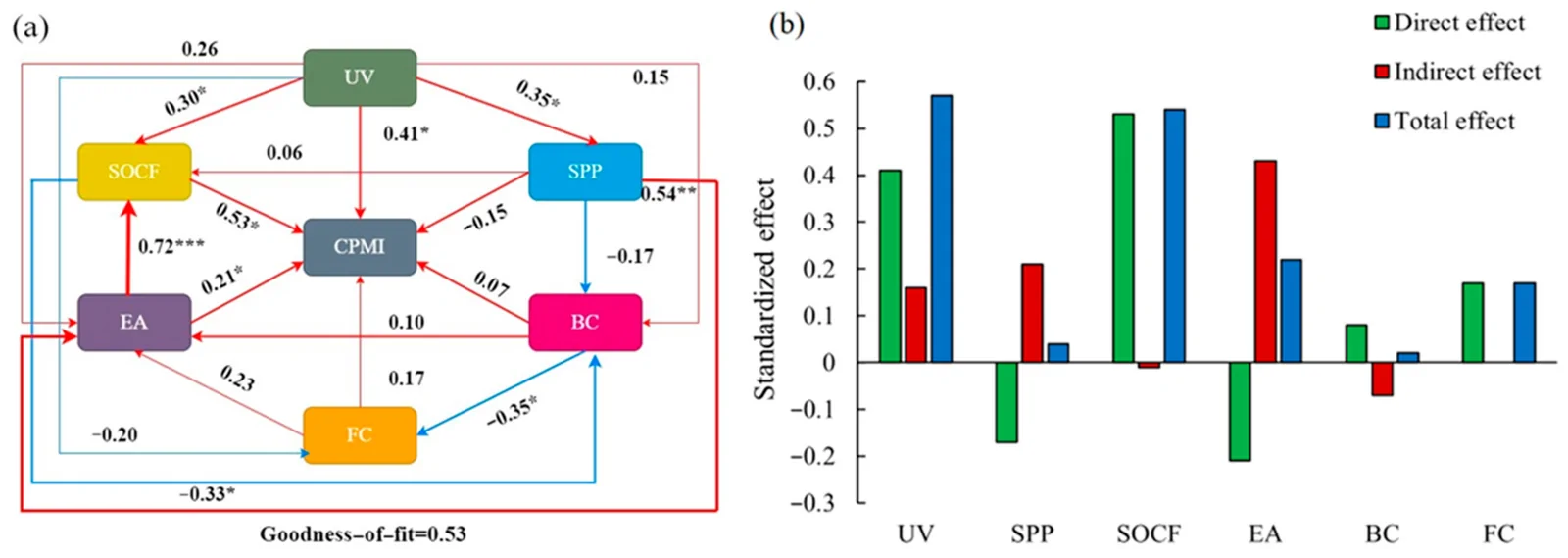
Partial least squares path modeling (PLS-PM) illustrating the direct and indirect effects of different factors on the CPMI. (**a**) Structural equation model; (**b**) Standardized direct, indirect and total effects derived from the model. Latent-variable definitions: UV, understory vegetation; SOCF, soil organic carbon fractions; SPP, soil physicochemical properties; EA, enzyme activity; BC, bacterial community; FC, fungal community. The red and blue arrows represent the positive and negative paths, respectively; the numbers next to the arrows represent the path coefficients; and the line thickness indicates the relative size of the path coefficients. *: *p* < 0.05, **: *p* < 0.01, ***: *p* < 0.001.

The correlation analysis revealed that the enzyme activity exhibited highly significant correlations with POC, SOC, POXC, and MBC (*p* < 0.001), while showing significant correlations with PN and AK (*p* < 0.05; Figure 8). The bacterial community was obviously correlated with TN (*p* < 0.05). Partial least squares path modeling (PLS-PM) showed a goodness-of-fit (GOF) value of 0.53 for the proposed model, exceeding the commonly accepted threshold of 0.5 and indicating a good overall model fit. The model was used to evaluate the associations among understory vegetation, soil properties, labile organic carbon fractions, enzyme activities, and the CPMI (Figure 9).

As shown in Figure 9a, understory vegetation showed positive direct paths to labile organic carbon fractions (0.30) and soil physicochemical properties (0.35, *p* < 0.05). Understory vegetation also showed a significant positive direct association with the CPMI (direct path coefficient = 0.41, *p* < 0.05). After considering indirect effects through intermediate variables (0.16), the total effect of understory vegetation on the CPMI reached 0.57 (Figure 9b). The enzymatic activity was positively associated with organic carbon fractions (0.72, *p* < 0.01). Soil properties showed a significant positive association with enzyme activity (*p* < 0.05). The soil organic carbon fractions showed a significant positive direct path to the CPMI (0.53, *p* < 0.05), whereas enzyme activity had a substantial negative direct effect (−0.21, *p* < 0.05).

In Figure 9b, although enzyme activity showed a negative direct effect on the CPMI, it exhibited a positive indirect effect through the soil organic carbon fractions (0.43), resulting in a positive total effect (0.22). Considering both direct and indirect pathways, the total effects of latent variables on the CPMI followed the order: understory vegetation (UV, 0.57) > soil organic carbon fractions (SOCFs, 0.54) > enzyme activities (EAs, 0.22) > fungal communities (FC, 0.17) > soil physicochemical properties (SPP, 0.04) > bacterial communities (BC, 0.02).

## 3. Discussion

### 3.1. Impact of Net Harvesting on Soil Quality

Our study showed a significant increase in soil pH with prolonged net-harvesting duration (*p* < 0.05; Table 2). This phenomenon may be associated with the elimination of herbicide application in netting systems. Conventional harvesting methods require herbicide usage, while the use of herbicides was discontinued after net harvesting. Herbicide residues have been reported to potentially influence soil acidity through their degradation products [26], which may partly explain the observed pH differences. Furthermore, the toxic effects of herbicides inactivate beneficial microorganisms, disrupt microbial community balance, and exacerbate soil acidification. The expansion of understory vegetation biomass and litter accumulation under extended netting duration was associated with increased carbon inputs and lower soil bulk density (*p* < 0.05; Table 2), possibly through improvements in soil aggregate structure [27]. Additionally, key nutrient elements (N and K) reached their highest concentrations in Y7, suggesting that increased vegetation inputs may contribute to improved soil nutrient availability. As vegetation recovered, the modified soil physicochemical properties influenced both bioavailable substrates and microbial community structure, potentially influencing soil carbon decomposition processes and SOC stability [28]. Our study revealed soil C/N ratios of 15.31, 15.69, and 12.81 in Y3, Y6, and Y7, respectively (Table 3). When the soil C/N ratio exceeded 25, the organic matter accumulation rate exceeded the decomposition rate, while ratios between 12 and 16 indicated moderate decomposition [29]. This interpretation is applicable to most soils. Therefore, the soil carbon and nitrogen decomposition rates in our study area are relatively fast.

Labile organic carbon fractions in soil, characterized by small molecular size and rapid turnover, serve not only as direct reservoirs of readily available nutrients but also as primary drivers of soil nutrient cycling [6]. Traditional beat harvesting involves understory vegetation removal, which may expose soil to direct sunlight and contribute to the depletion of labile carbon pools. The highly reactive nature of labile organic carbon makes it particularly sensitive to vegetation changes [30]. In Y6 and Y7, increased understory vegetation biomass was associated with higher POXC content (Figure 2). POC, which is known for its high mobility in soils, is strongly influenced by vegetation litter and root distribution [31]. MBC plays a pivotal role in organic carbon decomposition and formation and represents the most vegetation-sensitive fraction [32]. Existing evidence suggests that higher litter quantity and quality correlate with accelerated decomposition rates and consequent MBC accumulation [33]. DOC, primarily derived from fresh litter and predominantly existing as humic acids, showed increased concentrations with increasing netting duration. With increasing harvesting duration, extended netting may facilitate litter fragmentation and the release of biodegradable organic matter and DOC [34]. Soil water loss in winter was reduced, slowing down the leaching and loss of soil DOC in winter. This may be the reason for the significant increase of DOC content in winter (*p* < 0.05; Figure 2).

### 3.2. Changes in Soil Enzyme Activities and Microbial Community Under Net Harvesting

Soil enzymes such as NAG, βG, and LAP serve as important indicators of soil quality and are closely associated with SOC mineralization and nutrient cycling [35]. Our results showed that NAG activity in Y7 was significantly higher than that in the other treatments (Figure 3). This enhancement likely stems from the improved soil health and nutrient cycling under netting management. Seasonal variability was also an important factor influencing soil enzyme activities. Higher enzyme activities were generally observed in summer than in winter, likely due to increased plant-derived carbon inputs, higher microbial biomass, and more favorable environmental conditions for microbial metabolism during the growing season [35]. Vegetation restoration creates favorable conditions for soil carbohydrase activity [36]. Plant roots accelerate the formation of cell wall components (cellulose, hemicellulose, and lignin), thereby stimulating carbohydrase-mediated decomposition [37]. Specifically, βG hydrolyzes cellobiose into glucose for microbial utilization, with its activity increasing proportionally to the cellulose input from litter decomposition. During summer, the 7-year netting treatment resulted in a 30.36% increase in soil SC activity compared with traditional beat harvesting (*p* < 0.05; Figure 3). Enhanced organic matter inputs may therefore increase the availability of carbon substrates and contribute to changes in enzyme activities associated with organic matter decomposition [38]. The correlation analysis indicated highly significant relationships between enzyme activity and MBC, SOC, POXC, and POC (*p* < 0.01; Figure 8). These findings suggested that carbon-cycle-related extracellular enzymes exhibited synergistic interactions. They comprehensively participated in organic matter decomposition and nutrient cycling. Furthermore, labile organic carbon fractions effectively regulated enzyme activity levels [39,40].

More diverse plant communities produced more litter and root exudates, prompting the soil microbial community to respond positively [41]. In our study, the soil bacterial communities were predominantly composed of *Proteobacteria*, *Acidobacteria*, *Firmicutes*, and *Actinobacteria* (Figure 4). *Proteobacteria*, representing one of the largest prokaryotic phyla and comprising the most well-known Gram-negative bacteria, includes numerous phototrophs, heterotrophs, and chemolithotrophs [42]. The copiotroph hypothesis posits that nutrient-rich environments favored copiotrophic groups (e.g., *Proteobacteria*), whereas oligotrophic groups (e.g., *Acidobacteria*) dominated nutrient-poor environments [43]. Similar to the results of our study, traditional beat harvesting showed higher abundances of *Acidobacteria* and *Verrucomicrobia*, whereas *Firmicutes* reached peak abundance in Y7 (Figure 4a). Compared with the traditional treatment, net-harvesting treatments exhibited higher numbers of bacterial network nodes and edges, suggesting differences in potential bacterial co-occurrence patterns and network structures among treatments (Figure 5). Increased vegetation biomass after net harvesting may enhance the diversity of organic substrates, such as lignin, proteins, and condensed tannins, thereby potentially influencing bacterial community associations [44]. However, co-occurrence network analysis provides insights into potential associations among microbial taxa rather than direct ecological interactions. Given the limited number of biological replicates (*n* = 3 per treatment), treatment-specific network inference has limited statistical robustness. Therefore, the network topological characteristics observed in this study should be interpreted as descriptive patterns rather than definitive ecological interactions. Future studies with larger sample sizes and temporal monitoring are needed to further validate these microbial association networks.

Substrate quality and quantity differentially influence microbial growth. Complex carbon substrates and increased loading rates preferentially stimulate fungal growth over bacterial growth because of their sensitivity to litterfall [45,46]. At the taxonomic level analyzed, soil fungal communities were dominated by *Ascomycota*, *Basidiomycota*, and an unresolved fungal lineage (GS01) (Figure 6a). Most members of the phylum *Ascomycota* are saprophytic fungi and act as important decomposers of soil organic matter. They can form symbiotic relationships with plants and improve their stress resistance and growth ability. In addition, some Ascomycetes have antagonistic effects that can effectively inhibit the growth of other harmful microorganisms and reduce the occurrence of pests and diseases in pecan trees. *Basidiomycota* primarily degrade lignocellulose, thereby facilitating nutrient cycling [47].

The relative abundance of Basidiomycota increased after net harvesting and reached the highest level in Y3, followed by a decline under longer-term treatments (Figure 6a). This distinct response may be associated with the transitional stage of vegetation development in Y3, when increased plant-derived residues and lignocellulosic substrates potentially provided favorable resources for lignocellulose-degrading fungi. Similarly, the temporary enrichment of GS01 in Y3 may reflect the response of unresolved fungal lineages to changes in soil environmental conditions and substrate availability. With prolonged net-harvesting duration, continuous vegetation development and organic matter accumulation may gradually reshape fungal community composition toward a more stable structure.

### 3.3. Effect of Net Harvesting on Soil Carbon Pool Management Index

Compared with SOC alone, the CPMI provided a more comprehensive and objective assessment of soil quality dynamics, fertility status, and management impacts [10]. PLS-PM analysis revealed that understory vegetation was positively associated with the CPMI (direct path coefficient = 0.41, *p* < 0.05; Figure 9a), with a total effect of 0.57 after incorporating indirect pathways. This relationship may be explained by the potential contribution of vegetation-derived inputs to both labile and stable carbon pools through changes in substrate availability, decomposition processes, and aggregate protection mechanisms. Prolonged netting duration significantly increased understory biomass (*p* < 0.05; Table 1), and elevated biomass may provide greater litter and root-derived inputs, potentially increasing microbial carbon availability and metabolic activity, which may contribute to SOC accumulation [48]. Soil physicochemical properties showed a negative direct association with the CPMI, although their total effect was weakly positive after considering indirect pathways (Figure 9b). BD decreased after netting, which may have improved soil aeration and habitat conditions for soil organisms, potentially influencing organic matter transformation processes. Soil pH, a critical regulator of microbial activity and element bioavailability, may influence microbial habitat conditions and carbon cycling processes. Concurrently, elevated nitrogen and phosphorus availability was associated with greater vegetation growth and may have contributed to enhanced carbon inputs (Figure 9a). According to the results, enzyme activity showed a negative direct association with the CPMI (−0.21) but a positive indirect effect through the soil organic carbon fractions (0.43), resulting in a positive total effect (0.22; Figure 9b). The indirect pathway involving enzyme activity suggests that vegetation-driven increases in substrate availability first stimulate microbial metabolism, which then enhances carbon turnover efficiency captured by the CPMI. Higher enzymatic activity was associated with organic matter mineralization and POXC variation, which is a key determinant of the CPMI [49]. This aligns with findings from managed plantation soils, where sucrase and peroxidase activities significantly influence the CPMI [1]. The fungal community showed a positive association with the CPMI (total effect = 0.17), whereas the bacterial community showed a weaker association (total effect = 0.02) (Figure 9a). This suggested that shifts in microbial community composition under netting may be associated with increased availability of labile carbon substrates and enhanced carbon transformation processes, thereby contributing to CPMI improvement. Moreover, the decrease in the C/N enzyme ratio indicates that the nitrogen-releasing enzyme required for unit carbon acquisition is reduced, which can reduce carbon mineralization and respiration loss, thus indirectly boosting SOC storage [50]. These results highlight the potential role of net harvesting in improving soil carbon management and may contribute to sustaining long-term ecosystem productivity and resilience.

While this study provides significant insights, there are methodological limitations that must be acknowledged. Future studies should investigate the abundance and composition of microbial functional genes involved in soil carbon cycling to further clarify the mechanisms underlying the observed relationships between microbial processes and the CPMI. Therefore, the relationships identified by PLS-PM should be interpreted as potential ecological linkages rather than definitive causal pathways. Vegetation improvement and soil carbon accumulation may form a positive feedback loop, in which vegetation inputs enhance soil carbon processes while improved soil conditions further support vegetation development.

Nevertheless, this study has several limitations. Because different net-harvesting durations were established in different calendar years, the chronosequence approach may partially confound management duration with year-specific climatic variability and historical management conditions. In addition, the single-site design with three blocks per treatment limits the generalizability of our findings to other hickory plantations. Furthermore, although herbicide application was discontinued after the establishment of net-harvesting treatments, historical herbicide use in control plots represents an additional factor that may have influenced soil ecological processes. Multi-site experiments and longer-term monitoring are needed to further validate the ecological effects of net harvesting.

## 4. Materials and Methods

### 4.1. Study Sites

The *C. cathayensis* plantation in our study is located in Wucun Village, Taiyang Town, Lin’an District, Hangzhou City, Zhejiang Province, China (30°03′02″ N, 119°08′54″ E). The region belongs to the subtropical monsoon climate zone, with a mean annual temperature of 17.2 °C, average annual precipitation of 1613.9 mm, sunshine duration of 1837.9 h, and a frost-free period of 237 days. The study area is characterized by slate-derived Ferralsols according to the Food and Agriculture Organization (FAO) soil classification system [51], with acidic surface soils (0–20 cm) predominantly having a silt loam texture. The *C. cathayensis* plantation (40–50 years old) occupies slopes of 25–35° at an elevation of 300–340 m, with a stand density of 180–220 trees ha^−1^ and a mean diameter at breast height (DBH) of 22.35–26.57 cm.

The *C. cathayensis* plantation investigated in this study was a monospecific plantation, in which *C. cathayensis* was the only tree species and no other tree or shrub species occurred in the stands. The understory plant community was therefore mainly composed of herbaceous species. The dominant understory species included *Oplismenus undulatifolius*, *Lolium perenne*, *Vigna vexillata*, *Torilis scabra*, *Clinopodium gracile*, *Youngia erythrocarpa*, *Ophiopogon japonicus*, and *Elymus kamoji*, among others (Table S1). Thus, the vegetation structure of the studied plantation consisted primarily of a single-tree-species canopy and an herbaceous understory community.

### 4.2. Experimental Design

In 2018, three *C. cathayensis* forest plots with similar site conditions were chosen as three blocks. These plots were treated with 20% glyphosate aqueous solution from May to June each year to remove weeds. In 2019, one-fifth of each block was designated for the net treatment, forming Y7 (7 years of net harvesting). In 2020, an additional one-fifth of each block was netted, forming Y6 (6 years of net harvesting). In 2022, the same procedure was repeated, forming Y3 (3 years of net harvesting). In 2023, another one-fifth area was netted, forming Y2 (2 years of net harvesting). The remaining one-fifth area was set as the control (CK).

The netted plots allowed natural understory grass growth without manual weeding. A total of 15 plots were included, each measuring 50 m × 50 m. Uniform management practices, including water supply, fertilization, harvesting, and pest control, were implemented across all plots. Compound fertilizer (N:P_2_O_5_:K_2_O = 15:11:12) was applied at a rate of 400 kg·ha^−1^ in April and August annually for each plot.

All plots were located within the same hickory plantation and had similar stand age, soil conditions, topography, and previous management history before the implementation of net-harvesting treatments. Therefore, the initial conditions among plots were assumed to be comparable.

### 4.3. Aboveground Vegetation Collection and Determination

In June 2024, three 1 m × 1 m herbaceous quadrats were randomly established in each netting plot to record species names, coverage, and height of herbaceous plants. All aboveground herbaceous biomass within quadrats was collected to calculate VB. Fresh aboveground herbage was weighed, oven-dried at 105 °C for 30 min, then dried at 65 °C for 48 h. The dried material was ground through a 0.15 mm sieve, and the VC and VN contents were determined using an elemental analyzer (rapid CS cube, Elementar, Langenselbold, Germany) [52].

### 4.4. Soil Sample Collection and Determination

#### 4.4.1. Soil Sample Collection

During January (winter) and July (summer) of 2024, soil samples (0–20 cm depth) were collected from each plot following an “S”-shaped sampling pattern. For each plot, five subsamples were combined into a single mixed sample. Each treatment was repeated three times, that is, a total of 15 mixed samples were collected. Every mixed sample was divided into two aliquots: one was air-dried for physicochemical analysis, and the other was stored at 4 °C for the determination of labile organic carbon fractions and enzyme activities. Subsamples used for microbial community analysis were stored at −80 °C until DNA extraction.

#### 4.4.2. Soil Sample Determination

The air-dried soil samples were sieved through a 2 mm nylon mesh. A subsample of each soil was further ground in an agate mortar to pass through a 0.149 mm nylon sieve for subsequent analysis. Soil physicochemical properties were determined according to the methods described by Bao [53] and Xie et al. [54]. BD was determined using the cutting ring method. Soil pH was measured at a soil–water ratio of 1:2.5 (*w*/*v*). AN was determined using the alkaline hydrolysis diffusion method. AK was extracted with ammonium acetate and measured by flame photometry. AP was extracted with sodium bicarbonate and analyzed spectrophotometrically. TN was determined by the semi-micro Kjeldahl method. SOC was measured via the potassium dichromate-sulfuric acid oxidation method with external heating.

The fresh soil samples were sieved through a 2 mm nylon mesh. POC was isolated by sodium hexametaphosphate dispersion. POXC was determined by potassium permanganate oxidation. MBC was measured using chloroform fumigation–extraction. A k_EC_ value of 0.45 was used for microbial biomass carbon calculation. DOC was extracted with deionized water and quantified using a TOC analyzer (TOC-VCPH, Shimadzu, Kyoto, Japan) [55,56]. The activities of β-1,4-N-acetylglucosaminidase (NAG), β-glucosidase (βG), leucine aminopeptidase (LAP), sucrase (SC), and urease (UE) were determined using a fluorometric assay (Microplate Reader, Synergy™ H1, BioTek, Winooski, VT, USA) [57].

Genomic DNA was extracted from samples using the CTAB method. DNA concentration and purity were assessed via 1% agarose gel electrophoresis. Specific regions (ITS2) of soil fungal DNA were amplified using appropriate primers (e.g., ITS3-2024F and ITS4-2409R). Soil bacterial DNA was analyzed by PCR amplification of V3-V4 variable region with 341F and 806R primers. PCR products were screened by 2% agarose gel electrophoresis, and samples exhibiting a bright target band (400–450 bp) were selected for further analysis. The mixed PCR products were purified using a Qiagen Gel Extraction Kit (Qiagen, Hilden, Germany), and the TruSeq^®^ DNA PCR-Free SamPle PreParation Kit (Illumina, San Diego, CA, USA) was used to construct the sequencing library. Finally, the Illumina HiSeq2500 Platform was used for sequencing.

A two-step thermal cycling protocol was employed: initial denaturation at 95 °C for 5 min, followed by 40 cycles of 95 °C for 15 s and 60 °C for 34 s, with a final extension at 95 °C for 15 s. Melting curve analysis was performed after amplification to verify the specificity of PCR products. Raw sequencing reads were processed using the QIIME2 DADA2 pipeline for quality filtering, trimming, denoising, paired-end merging, and chimera removal. Amplicon sequence variants (ASVs) were inferred using DADA2. Taxonomic classification was performed using the Greengenes database for bacterial 16S rRNA gene sequences and the UNITE database for fungal ITS sequences. Non-target sequences were removed using the QIIME2 feature-table plugin. For fungal communities, unresolved taxonomic lineages lacking formally described taxonomic assignments were retained as unresolved fungal groups rather than assigned to established taxonomic categories.

Sequencing quality and depth were assessed based on the number of non-chimeric reads, observed ASV richness, and rarefaction curves. Rarefaction curves were generated based on observed ASV numbers to evaluate whether sequencing depth was sufficient to capture microbial diversity before downstream community analyses.

Principal component analysis (PCA) was performed based on phylum-level relative abundance data to visualize overall patterns of microbial community composition and differences among treatments.

### 4.5. Calculations

According to the results of the plot survey, the species richness, the Shannon–Wiener index, and the Simpson index were calculated using the following equations (Equations (1)–(3)):

(1) $$R^{\prime }=S$$

(2) $$H^{\prime }=-\sum_{i=1}^{S}P_{i}InP_{i}$$

(3) $$D_{S}=1-\sum_{i=1}^{S}P_{i}^{2}$$

where R′ is the species richness; H′ is the Shannon–Weiner index; Ds is the Simpson index; Pi is the probability of the i-th plant appearing in the plot; and S is the total number of species.

The calculation formula of the soil carbon pool management index (CPMI) is as follows:

(4) $$CPI=\frac{SOC\;sample}{SOC\;reference}$$

(5) NLOC=SOC sample−LOC sample

(6) $$L=\frac{LOC\;sample}{NLOC}$$

(7) $$LI=\frac{L}{LO}$$

(8) CPMI=CPI×LI×100

where CPI represents the carbon pool index, calculated as the ratio of SOC in each treatment to that in CK (SOC_reference). L represents carbon pool lability, and LI represents the lability index. Following the concept of the CPMI proposed by Blair et al. [58] (Equations (4)–(8)), LOC represents the composite labile organic carbon pool calculated from multiple carbon fractions, including POC, POXC, DOC, and MBC. Before calculation, all carbon fractions were converted into the same unit (mg kg^−1^), and LOC was obtained by summing these standardized carbon components. These fractions were converted into consistent units before calculation. NLOC represents the non-labile organic carbon pool, calculated as the difference between total SOC and LOC. L0 represents the reference lability calculated from the CK treatment. Since CK served as the reference treatment, its CPMI value was normalized to 100 by definition.

Total enzyme activity (TEA) was calculated according to the following equation:

(9) $$TEA=\sum_{i=1}^{n}\frac{x_{i}}{\bar{x}}$$

where xi represents the measured activity value of the i-th enzyme in a tested soil sample; $\bar{x}$ is the average activity value of the corresponding enzyme across all samples; *n* equals the total number of the soil enzyme types determined (*n* = 5).

### 4.6. Statistical Analysis

All statistical analyses were performed using SPSS 25.0 (IBM, Armonk, NY, USA). Prior to ANOVA, data were tested for normality and homogeneity of variance, and logarithmic transformations were applied when necessary [59]. Differences among treatments were analyzed using analysis of variance (ANOVA) based on a randomized complete block design (RCBD), followed by Duncan’s multiple range test at α = 0.05. When data were collected repeatedly across different seasons from the same plots, a two-way repeated-measures ANOVA was performed, with treatment as the between-subject factor and season as the within-subject factor. The main effects of treatment, season, and their interaction were evaluated.

Principal component analysis (PCA) was performed as an exploratory ordination approach to visualize overall patterns of microbial community variation among treatments based on phylum-level relative abundance profiles. Microbial co-occurrence networks were built with the igraph package in R 4.3.2, retaining only bacterial and fungal taxa with relative abundances > 0.1% and > 0.01%, respectively, at the phylum level [60]. Taxa were aggregated at the phylum level to reduce network complexity and improve interpretability given the limited number of biological replicates. Spearman correlation coefficients were calculated among dominant microbial taxa, and associations with |r| ≥ 0.5 and *p* < 0.05 were retained as network edges. Network visualization was performed using Gephi (version 0.9.2). Topological properties (node count, edge number, and positive correlation ratio) were calculated to evaluate network complexity [61]. Mantel tests assessed correlations among vegetation characteristics, soil physicochemical properties, the CPMI, enzyme activities, and microbial communities [19]. Partial least squares path modeling (PLS-PM) quantified the direct and indirect effects of these factors on the CPMI [62,63].

PLS-PM was conducted to evaluate the direct and indirect relationships among understory vegetation (UV), soil physicochemical properties (SPPs), soil organic carbon fractions (SOCFs), enzyme activities (EAs), bacterial communities (BCs), fungal communities (FCs), and the CPMI. The manifest indicators and their outer loadings for each latent variable are provided in Supplementary Table S4. Model reliability was assessed using composite reliability, while the explanatory power of endogenous constructs was evaluated using R^2^ values. The significance of path coefficients was tested using bootstrap resampling with 5000 iterations. In the PLS-PM framework, SOCF was incorporated as a composite indicator representing variations in soil carbon pool characteristics based on SOC, POXC, POC, and MBC, rather than as a direct mathematical component used for the CPMI calculation.

## 5. Conclusions

Net harvesting management improved the biomass and nutrient content of understory vegetation. Increased vegetation biomass may have contributed to greater litter inputs and root-derived carbon inputs, which were associated with changes in soil physicochemical properties and soil structure. The favorable soil environmental conditions were accompanied by increased activities of five key enzymes, including NAG, βG, LAP, SC, and UE. Furthermore, vegetation-derived inputs may have increased the availability of labile carbon substrates, which were associated with shifts in microbial community structure and functional potential related to nutrient cycling. Under net harvesting, the increases in SOC and its active fractions (*p* < 0.05) were associated with higher CPMI values. Overall, our findings suggest that long-term net harvesting may contribute to the sustainability of *C. cathayensis* plantations through coordinated interactions among understory vegetation development, soil carbon processes, enzyme activities, and microbial community dynamics, which together may promote soil carbon and nutrient cycling.

## Figures and Tables

**Table 1:** Descriptive statistics of vegetation under different treatments.

Treatment	VB (g·m^−2^)	VC (g·kg^−1^)	VN (g·kg^−1^)	VC/VN
CK	43.85 ± 13.15 c	397.77 ± 85.02 b	21.78 ± 3.31 a	18.58 ± 4.98 b
Y2	72.53 ± 12.51 c	436.82 ± 132.73 ab	21.90 ± 15.94 a	19.83 ± 0.59 ab
Y3	163.52 ± 25.36 b	560.51 ± 58.12 a	21.50 ± 12.89 a	26.60 ± 6.19 a
Y6	203.69 ± 17.64 a	507.59 ± 39.11 ab	27.46 ± 0.74 a	18.48 ± 1.26 b
Y7	172.28 ± 20.15 ab	486.00 ± 51.86 ab	21.55 ± 2.09 a	22.79 ± 4.01 ab

Mean ± SD, *n* = 3. Values within a column and treatment group followed by different letters are significantly different (*p* < 0.05). VB: vegetation biomass, VC: vegetation total carbon, VN: vegetation total nitrogen.

**Table 2:** Descriptive statistics of soil physical and chemical properties under different treatments.

	Treatment	pH	BD (g·cm^−3^)	AN (mg·kg^−1^)	AP (mg·kg^−1^)	AK (mg·kg^−1^)
Summer	CK	6.44 ± 0.51 c	1.27 ± 0.09 a	200.00 ± 20.95 b	13.67 ± 1.52 ab	191.33 ± 21.39 b
	Y2	6.86 ± 0.26 b	1.21 ± 0.03 a	247.33 ± 39.88 b	10.50 ± 1.26 b	193.30 ± 17.62 b
	Y3	6.78 ± 0.03 b	1.12 ± 0.04 b	255.33 ± 60.04 b	13.01 ± 2.61 ab	155.33 ± 26.56 b
	Y6	6.85 ± 0.24 b	1.17 ± 0.06 b	351.67 ± 15.04 a	12.36 ± 3.85 ab	186.67 ± 24.58 b
	Y7	7.20 ± 0.17 a	1.11 ± 0.09 b	328.33 ± 32.39 a	16.18 ± 6.15 a	203.00 ± 25.24 a
Winter	CK	5.57 ± 0.12 d	1.17 ± 0.10 a	285.33 ± 46.14 a	7.67 ± 2.66 a	114.33 ± 17.61 a
	Y2	6.77 ± 0.24 ab	1.10 ± 0.06 a	202.67 ± 16.20 b	6.75 ± 0.52 b	77.00 ± 14.00 b
	Y3	6.09 ± 0.17 c	1.07 ± 0.05 a	280.33 ± 32.62 a	7.00 ± 1.85 ab	105.00 ± 7.00 a
	Y6	6.26 ± 0.22 bc	1.06 ± 0.08 a	183.33 ± 17.93 b	6.39 ± 1.76 b	102.67 ± 10.69 a
	Y7	6.86 ± 0.50 a	0.94 ± 0.10 a	222.00 ± 47.15 ab	5.95 ± 0.13 c	121.33 ± 10.69 a

Values within a column and treatment group (summer or winter) followed by different letters are significantly different (*p* < 0.05).

**Table 3:** Soil organic carbon, total nitrogen, and the C/N ratio under different treatments.

	Treatment	SOC (g·kg^−1^)	TN (g·kg^−1^)	C/N
Summer	CK	12.19 ± 1.47 c	1.59 ± 0.32 b	7.71 ± 1.22 b
	Y2	15.20 ± 3.18 bc	1.77 ± 0.37 b	8.60 ± 0.45 b
	Y3	21.37 ± 0.12 b	1.45 ± 0.33 b	15.31 ± 3.94 a
	Y6	32.40 ± 3.11 a	2.10 ± 0.43 a	15.69 ± 1.94 a
	Y7	31.27 ± 0.51 a	2.45 ± 0.14 a	12.81 ± 0.56 a
Winter	CK	17.96 ± 3.83 b	2.28 ± 0.30 a	7.87 ± 1.15 b
	Y2	22.85 ± 3.34 ab	2.41 ± 0.22 a	9.50 ± 1.31 ab
	Y3	20.29 ± 2.08 ab	2.24 ± 0.43 a	9.16 ± 0.91 ab
	Y6	25.56 ± 6.11 a	2.31 ± 0.30 a	11.15 ± 2.67 a
	Y7	24.22 ± 2.10 ab	2.48 ± 0.13 a	9.79 ± 1.02 ab

Values within a column and treatment group (summer or winter) followed by different letters are significantly different (*p* < 0.05).

**Table 4:** Soil carbon pool management index under different treatments.

	Treatment	CPI	L	LI	CPMI
Summer	CK	1.00 ± 0.00 c	0.40 ± 0.05 a	1.00 ± 0.00 a	100.00 ± 0.00 b
	Y2	1.28 ± 0.36 bc	0.41 ± 0.10 a	1.01 ± 0.24 a	123.78 ± 12.86 ab
	Y3	1.79 ± 0.36 b	0.39 ± 0.09 a	0.97 ± 0.21 a	171.91 ± 24.96 a
	Y6	2.67 ± 0.30 a	0.24 ± 0.02 b	0.61 ± 0.12 a	165.35 ± 41.94 a
	Y7	2.62 ± 0.50 a	0.26 ± 0.01 b	0.64 ± 0.07 a	172.09 ± 47.69 a
Winter	CK	1.00 ± 0.00 a	0.18 ± 0.01 ab	1.00 ± 0.00 ab	100.00 ± 0.00 c
	Y2	1.33 ± 0.40 a	0.16 ± 0.04 b	0.86 ± 0.25 b	107.28 ± 8.29 bc
	Y3	1.15 ± 0.14 b	0.22 ± 0.05 ab	1.23 ± 0.22 ab	143.06 ± 42.39 b
	Y6	1.43 ± 0.26 a	0.18 ± 0.03 ab	1.01 ± 0.21 ab	141.16 ± 9.63 b
	Y7	1.40 ± 0.38 a	0.26 ± 0.06 a	1.41 ± 0.37 a	188.26 ± 5.39 a

Values within a column and treatment group (summer or winter) followed by different letters are significantly different (*p* < 0.05). CPI is the carbon pool index; L is the lability of carbon pool; LI is the lability index.

**Table 5:** Topological attributes of soil bacterial community network complexity under different treatments.

Topological Characteristic	CK	Y2	Y3	Y6	Y7
Total nodes	48.00	47.00	62.00	66.00	65.00
Total connections	235.00	381.00	376.00	491.00	726.00
Positive correlation (%)	54.89	53.28	56.91	50.31	50.55
Average	9.79	16.21	12.13	14.88	22.34
Graph density	0.21	0.35	0.20	0.23	0.35
Modularity	0.70	0.26	0.76	0.59	0.31

**Table 6:** Topological attributes of soil fungal community network complexity under different treatments.

Topological Characteristic	CK	Y2	Y3	Y6	Y7
Total nodes	30.00	43.00	63.00	67.00	74.00
Total connections	98.00	178.00	478.00	529.00	641.00
Positive correlation (%)	83.67	72.47	65.69	65.22	55.54
Average	6.53	8.29	15.18	15.79	17.32
Graph density	0.23	0.20	0.25	0.24	0.24
Modularity	0.64	0.68	0.61	0.61	0.64

## Data Availability

The datasets generated for this study can be found in the NCBI Sequence Read Archive (SRA) database under the BioProject ID PRJNA1506087. Additional data are available on request from the corresponding author.

## Supplementary Materials

The following supporting information can be downloaded at: https://www.mdpi.com/article/10.3390/plants15172589/s1. Figure S1. Linear relationship between soil labile organic carbon fractions and total organic carbon under different treatments. Figure S2. Rarefaction curves of bacterial and fungal communities based on observed ASV numbers. Table S1. Top 5% of understory vegetation richness in *C. cathayensis* plantations. Table S2. Shannon-Wiener and Simpson diversity indices of understory vegetation under different net-harvesting durations. Table S3. Two-way repeated-measures ANOVA on the effects of treatment and season on soil physicochemical properties and enzyme activities. Table S4. Manifest indicators, outer loadings, and reliability assessment of latent constructs used in the PLS-PM analysis.
