# Supplementary material for: Long-Term Net Harvesting Is Associated with Elevated Soil Carbon Pool Management Index in Chinese Hickory Plantations
Source: Plants (Basel). 2026 Aug 25;15(17):2589. doi: 10.3390/plants15172589 (PMC13567646; doi:10.3390/plants15172589)
Supplement: Supplementary file 1 [file plants-15-02589-s001.zip › plants-4439456-supplementary.pdf]

Supplementary material of Long-term net-harvesting is associated with elevated soil carbon pool management index in Chinese hickory plantations

Supplementary Figure S1. Linear relationship between soil labile organic carbon fractions and total organic carbon under different treatments

Supplementary Figure S2. Rarefaction curves of bacterial and fungal communities based on observed ASV numbers.

Supplementary Table S1. Top 5% of understory vegetation richness in *C. cathayensis* plantations

Supplementary Table S2. Shannon-Wiener and Simpson diversity indices of understory vegetation under different net-harvesting durations

Supplementary Table S3. Two-way repeated measures ANOVA on the effects of treatment and season on soil physicochemical properties and enzyme activities

Supplementary Table S4. Manifest indicators, outer loadings, and reliability assessment of latent constructs used in the PLS-PM analysis

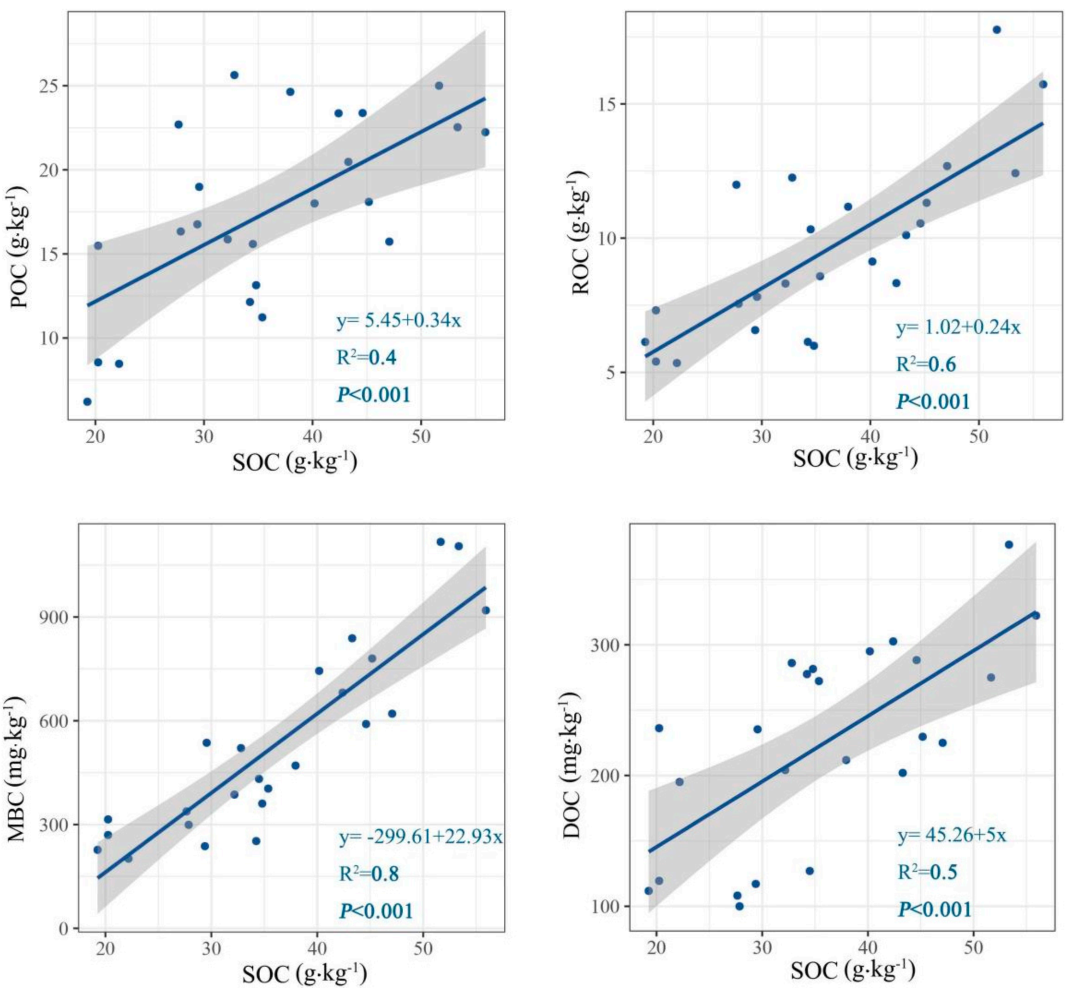

Supplementary Figure S1 Linear relationship between soil labile organic carbon fractions and total organic carbon under different treatments.

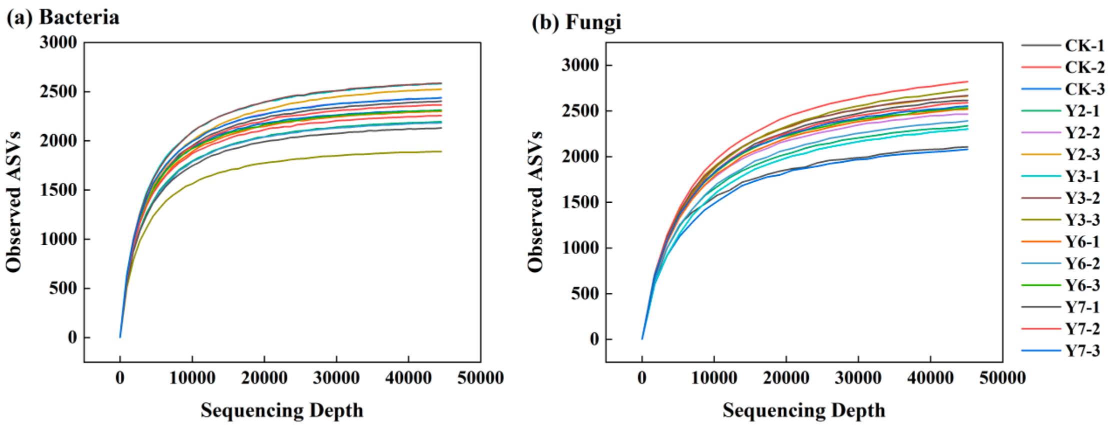

Supplementary Figure S2 Rarefaction curves of bacterial and fungal communities based on observed ASV numbers.

Supplementary Table S1

Top 5% of understory vegetation richness in *C. cathayensis* plantations

Vegetation Form	CK	Y2	Y3	Y6	Y7
Oplismenus undulatifolius	16.67	——	——	——	——
Lolium perenne	11.00	——	13.00	——	——
Vigna vexillata	7.00	——	——	——	——
Erigeron annuus	4.70	——	——	——	——
Sibirotrisetum bifidum	4.50	——	——	——	——
Torilis scabra	——	15.30	——	23.50	——
Clinopodium gracile	——	10.50	——	18.00	——
Viola diffusa	——	10.00	——	——	——
Sinosenecio oldhamianus	——	8.50	——	25.00	——
Youngia erythrocarpa	——	7.50	6.00	8.50	——
Poa acroleuca	——	——	7.50	——	——
Ophiopogon japonicus	——	——	7.20	6.30	6.33
Elymus kamoji	——	——	6.50	7.20	7.00
Nanocnide lobata	——	——	——	——	67.67
Ceratium glomeratum	——	——	——	——	9.00
Metathelypteris laxa	——	——	——	——	6.00

Supplementary Table S2

Shannon-Wiener and Simpson diversity indices of understory vegetation under different net-harvesting durations

Treatment	Shannon	Simpson
CK	2.42±0.28a	0.85±0.07a
Y2	1.76±0.24a	0.74±0.07a
Y3	2.10±0.44a	0.79±0.15a
Y6	1.66±0.16a	0.73±0.07a
Y7	1.73±0.39a	0.70±0.69a

Values within a column and treatment group followed by different letters are significantly different (P < 0.05).

Supplementary Table S3

Two-way repeated measures ANOVA on the effects of treatment and season on soil physicochemical properties and enzyme activities

Indicator	Source	F_value	P_value	Significance
pH	Treatment	5.42	0.0040	**
	Season	9.78	0.0053	**
	Treatmen×Season	0.68	0.6133	
Bulk density (BD)	Treatment	1.25	0.3231	
	Season	2.87	0.1059	
	Treatmen×Season	0.65	0.6318	
Available nitrogen (AN)	Treatment	0.26	0.8981	
	Season	6.56	0.0186	*
	Treatmen×Season	6.39	0.0018	**
Available phosphorus (AP)	Treatment	0.46	0.7629	
	Season	8.58	0.0083	**
	Treatmen×Season	0.04	0.9968	
Available potassium (AK)	Treatment	1.74	0.1811	
	Season	54.90	<0.0001	***
	Treatmen×Season	1.47	0.2491	
Soil organic carbon (SOC)	Treatment	15.19	<0.0001	***
	Season	1.25	0.2772	
	Treatmen×Season	4.03	0.0148	*
Total nitrogen (TN)	Treatment	1.43	0.2610	
	Season	24.40	<0.0001	***
	Treatmen×Season	4.88	0.0065	**
Carbon to nitrogen ratio (C/N)	Treatment	5.78	0.0029	**
	Season	12.54	0.0021	**
	Treatmen×Season	4.25	0.0119	*
Dissolved organic carbon (DOC)	Treatment	10.29	<0.0001	***
	Season	56.28	<0.0001	***
	Treatmen×Season	2.74	0.0573	
Particulate organic carbon (POC)	Treatment	2.54	0.0715	
	Season	0.15	0.7031	
	Treatmen×Season	0.41	0.8031	
Microbial biomass carbon (MBC)	Treatment	13.07	<0.0001	***
	Season	23.96	<0.0001	***
	Treatmen×Season	2.47	0.0779	
Permanganate oxidizable carbon (POXC)	Treatment	38.58	<0.0001	***
	Season	22.36	<0.0001	***
	Treatmen×Season	7.16	<0.0001	***
Carbon pool management index (CPMI)	Treatment	4.43	0.0100	*
	Season	0.07	0.8020	
	Treatmen×Season	0.62	0.6525	
β-1,4-N-acetylglucosaminidase	Treatment	0.80	0.5422	

Indicator	Source	F_value	P_value	Significance
(NAG)	Season	20.09	<0.0001	***
	Treatmen×Season	1.32	0.2973	
β-glucosidase (βG)	Treatment	0.85	0.5087	
	Season	26.20	<0.0001	***
	Treatmen×Season	0.56	0.6914	
Leucine aminopeptidase (LAP)	Treatment	3.86	0.0175	*
	Season	0.03	0.8632	
	Treatmen×Season	0.07	0.9904	
Sucrase (SC)	Treatment	27.19	<0.0001	***
	Season	106.51	<0.0001	***
	Treatmen×Season	13.70	<0.0001	***
Urease (UE)	Treatment	5.68	0.0032	**
	Season	79.19	<0.0001	***
	Treatmen×Season	1.70	0.1891	
Total enzyme activities (TEA)	Treatment	0.516	0.7249	
	Season	0.468	0.502	
	Treatmen×Season	0.54	0.7083	

Treatment includes CK, Y2, Y3, Y6, and Y7 (different net-harvesting durations). Season refers to summer and winter. Treatment × Season indicates the interaction effect between treatment and season. *, **, and *** indicate significant differences at the $P < 0.05$, $P < 0.01$, and $P < 0.001$ levels, respectively.

Supplementary Table S4

Manifest indicators, outer loadings, and reliability assessment of latent constructs used in the PLS-PM analysis

Latent construct	Manifest indicators	Outer loading	Composite reliability (DG.rho)	AVE	R^2
Understory Vegetation (UV)	Vegetation biomass carbon	0.994	0.993	0.974	—
	Vegetation phosphorus content	0.999			
	Vegetation nitrogen content	0.972			
	Vegetation C/N ratio	0.976			
Soil physicochemical properties (SPP)	Total nitrogen (TN)	0.888	0.912	0.722	0.232
	Available nitrogen (AN)	0.692			
	Available phosphorus (AP)	0.894			
	Available potassium (AK)	0.909			
Soil organic carbon fractions (SOCF)	Soil organic carbon (SOC)	0.912	0.944	0.808	0.894
	Readily oxidizable carbon (ROC)	0.917			
	Particulate organic carbon (POC)	0.847			
	Microbial biomass carbon (MBC)	0.920			
Enzyme activity (EA)	Sucrase (SC)	0.924	0.971	0.847	0.846
	Urease (UE)	0.899			
	β-1,4-N-acetylglucosaminidase (NAG)	0.916			
	β-glucosidase (βG)	0.975			
	Leucine aminopeptidase (LAP)	0.838			
Bacterial community (BC)	Bacterial Chao1 richness	0.847	0.902	0.823	0.781
	Bacterial Shannon diversity	0.951			
Fungal community (FC)	Fungal Shannon diversity	0.934	0.954	0.912	0.401
	Fungal Simpson diversity	0.972			
Carbon pool management index	CPMI	1.000	1.000	1.000	0.808

CPMI was specified as a single-item endogenous construct.
